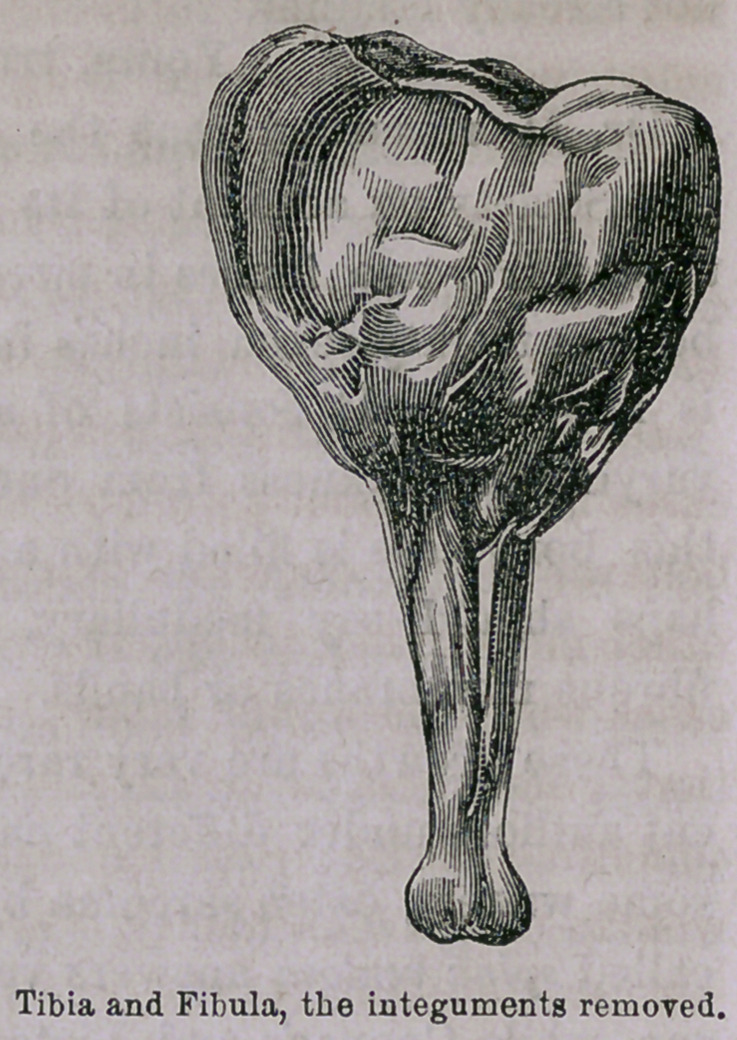# Abstract of the Proceedings of the Buffalo Medical Association

**Published:** 1865-03

**Authors:** Joseph A. Peters

**Affiliations:** Secretary


ART. II.—*Abstract of the Proceedings of the Buffalo Medical Association.*

Tuesday Evening, February 7th.

Association met in the new room, pursuant to adjournment, the President, Dr. Samo, in the Chair. Present, Drs. White, Lockwood, Miner, Strong, Congar, Cronyn, Jansen, Wetmore, Peters, and Burger.

The President wished to congratulate the Association on the new room secured for its future use. It now had a home, a long lease, and low rent, and there was no good reason why it should not go on improving. Our monthly meetings should be made more profitable and more attractive. Nothing sinister or selfish could conduce to that end, hence all should labor with a single eye to improvement.

The minutes of the last meeting were read and approved.

*Dr. Miner* presented some specimens he had recently removed by operation.

The 1st was a remarkable bone tumor which had formed in the upper portion of the tibia. The following was the history of the case, which had been furnished by Dr. Durbarow of Corfu, who had charge of the patient, and through whose favor Dr. M. had made the operation for removal, and been allowed to retain the specimen:

"Corfu, February 11, 1865.

*Dear Doctor:*—Your patient in Pembroke, Mr. G., reports that the commencement of his trouble was in this wise: 'In 1848, while living in Mississippi, and carrying some heavy timber, he slipped, falling upon his knee and striking it near the insertion of the *ligamentum patella.* Supposed that he had sprained the knee; was at that time confined a month, and then went to work again, but soon was obliged to abandon it. In the spring of 1849 he returned to his home in Pembroke, at which time there was a hard tumor near the place of injury, about an inch in diameter. He consulted Dr. Frank H. Hamilton, who thought it fungus hæmatodes, and advised amputation. Was treated for a year by various physicians, and then discontinued all treatment until he came under my care, about six weeks before you made the amputation. When I first saw him he was greatly reduced in flesh and strength. The growth had been gradual from the first. It is now three weeks since the operation, and it is nearly healed up; he is in good spirits and doing well. There is nothing else in the case with which you are not already familiar.'

Yours, truly, J. DURBORAW."

Dr. M. remarked that the specimen had been presented before the Society on account of its variety and interest. The leg measured thirty-two inches in circumference before amputation. The bone is twenty-seven inches in circumference after the integument is removed. It consists of a pretty firm, bony wall externally, varying in thickness from one or two lines to an inch. Internally this bony case is filled with a firm medullary or fibrous mass, perhaps should say medullary, separated in various directions by fibrous membranes or bands.

These growths are very rare, and have been described by different authors under different names; formerly called *spina ventosa* by some writers, osteo sarcoma by others. The description of disease called *spina ventosa* answers very well for this case as given by Gibson, while Cooper's and Liston's descriptions do not answer at all. Paget, places such disease under the head of myeloid tumor of bone, and that is quite expressive of the nature of the disease. Medullary tumor of bone is a sufficiently correct name, while the nosological classification of bone tumors is as yet quite imperfect. The causes are undetermined, though injury as in this case, is often regarded as the primary or exciting cause. Much speculation has also been made as to how this condition is brought about in bone, some supposing that the bony wall is expanded by pressure from within: others maintaining that the bone wall is secreted or formed from the periosteum, while the original bone is carried away by absorption. That there is great deposit or formation of bone tissue in this case, admits of no doubt; the bone wall is thick and firm, in many parts an inch in thickness, while in others it is very thin, or even in one or two places entirely wanting. The appearance of the external surface upon dissection, indicated the continual growth of bone; in many places unattached bony material was observed surrounding the tumor, and where the bone wall was thinnest, it appeared as if ossification was still going on upon the outer surface, the tissues being part bony, and part fibrous. The periosteum was not easily traced, or was changed in appearance and structure, being unlike that membrane as it is observed covering healthy bone.

Appearance of leg before amputation.

Tibia and Fibula, the integuments removed.

The 2d pathological specimen presented, was a cystic tumor, which Dr. Miner had that day removed from the neck of a patient at the General Hospital. It rested upon the carotid artery occupying one of the most important surgical regions. It was removed without great difficulty, as is the case with most of the cystic growths which appear in this region, but is presented as a some. what remarkable specimen, since attached to it, and removed with it is an enlarged gland, and between the tumor proper and gland, is an ossific deposit; so that we have cystic, glandular and bony tumor, united together, and removed as one mass. The gland was from the near region of the thyroid, was about the size of that gland, and a careless observer might suppose that the thyroid gland had been removed; it extended from the cystic growth and lay directly over or above the thyroid. As before remarked the bony deposit is between these two growths.

The 3d specimen is a melanoid tumor which grew from the orbit one year after the removal of the eye for melanotic disease. The patient was presented before the Society in the very commencement of the disease by Dr. Miner, and this tumor is now brought forward to complete this history. The opinion was then expressed and published in the transactions, that the disease was melanosis, but it took time to convince all, of such an unhappy view. The patient visited the New York Eye Infirmary, and after some delay and indecision, the surgeons concluded to adopt this view, and acted upon the advice long before given and published. The results of the case could not probably have been changed by earlier removal, and its re-removal has not been with the view that any great good would be accomplished; unmistakably malignant in its character and certainly fatal in its results.

*Dr. Lockwood* said he wished to be understood in advance of the cases he was about to relate, as having always been an entire disbeliever in the doctrine that gonorrhœa could be produced by any other means than by specific virus in illicit intercourse. The three cases which he would relate, however, had served to convince him that gonorrhœa or an affection not to be distinguished from it did sometimes arise from excessive connection, or connection with a woman of unclean habits, where no specific virus exists.

Case I.—Some ten or twelve years ago he was consulted by a worthy old couple living some fifteen or twenty miles in the country. Both were very fleshy, and madame not very cleanly in her personal habits; in short, rather nasty than otherwise. They lived a long way from any neighbors, were honest, simple folks. and the probabilities were very strong, apart from any assertions against either of them ever having indulged in illicit intercourse. On examining the husband he informed the Doctor that several times during the past few years he had had after intercourse, a slight degree of irritation and mucus discharge from the urethra, which had always disappeared in a few days on his taking a dose of Epsom salts, and paying attention to cleanliness. The present attack was one of, to all appearance, severe gonorrhœa in the inflammatory stage. The secretion of muco-purulent discharge was so great as to drop from the orifice of the urethra. There was also some slight chordee. He protested he had not been from home in months, and had had intercourse with no one but his wife. On examining the woman no symptoms of gonorrhœa were discoverable, but as has been stated her habits as regards cleanliness were very bad. She had had since the birth of her last child, (some ten years) a leucorrhœal discharge, which was at times accompanied with a good deal of irritation about the oulva and vagina. When her husband had last had iutercourse with her she had been working hard during the day, was not yet fairly through with her menses, and the discharge was worse than usual. She also denied any unfaithfulness to her marital duties. She was cured of her leucorrhœa by the use of cleanliness and proper remedies, and her husband put under treatment for gonorrhœa, with good results, though the case was somewhat tedious. They never had any more cause to complain of each other's conduct.

Case II—Occurred in the city. The husband solemnly declared he had never had gonorrhœa and had not been unfaithful. About four or five days after having connection with his wife was attacked with what appeared to be gonorrhœa. He consulted a physician, who pronounced it such, and assured him it could only have been contracted in one way, and that either he or his wife had been astray. He hurried home, charged his wife with unfaithfulness, and prepared for a separation. The wife declared her innocence, and having called in her mother and mother-in-law, Dr. Lockwood was sent for. The woman had no gonorrhœa, but had been recently confined, and had profuse lochial discharge, with some irritation of the vagina. Intercourse had taken place within ten days after her confinement, much against her inclinations. After some argument, and assuring the husband that there was not anything in his condition necessarily implying unfaithfulness on the part of his wife, he (Dr. L.) succeeded in restoring peace between them, and months afterwards they united in thanking him for his interference. It should be noted in this case that the disease developed in the husband yielded much more easily to remedies than in the former case.

Case III.—Also occurred in the city. The parties had been married about two years, and had no family. The man presented a well defined case of gonorrhœa, but, declaring his entire faithfulness, accused his wife of the opposite fault. It appeared from her statement that he was a man of strong animal propensities, and had indulged them to an excessive extent, paying no attention to his wife's condition. Her last menses had been quite excessive, and connection had taken place during their continuance. She also protested her entire innocence,

In conclusion, he (Dr. Lockwood) wished to say that he had brought this subject before the Society solely because he believed it to be an important one in its bearings upon the peace of families, and he firmly believed that, although very rarely met with, cases did sometimes occur, not to be distinguished from true gonorrhœa, which involved no *necessary* suspicion of illicit intercourse. He thoroughly believed in the truth of the statement made by the parties alluded to. It should be borne in mind that there was a moral evidence in such cases, which could not be given in any relation. Wished to hear the opinions of others on the subject.

*Dr. White* was very glad indeed that the subject of specific virus in gonorrhœa had been introduced here by Dr. Lockwood, as he considered it had a very important bearing many times in those sort of medico-legal investigations which physicians were often called on to make, where the family physician became, to a certain extent, judge and jury, and upon his dictum might depend the peace and happiness of the family. Virtue had often suffered under unjust imputations, because it was not more generally understood that simple gonorrhœa may result from contact with the irritating discharges of the vagina. Thought he had more often seen it produced by diseased discharges than by the menses. Referred to a monograph on the subject by Dr. A. K. Carter.

*Dr. Jansen* had been very incredulous on the subject, but had no doubt such cases did sometimes occur, and thought it important it should be understood. Had recently been called in a family where there were three small children—two boys and a girl. One of the boys had phimosis, one paraphimosis, and the girl vaginitis.

*Dr. Congar* was also glad the subject had been broached. Had seen a few such cases in his own practice. Its importance could not be overrated.

*Dr. Miner* had no doubt of the existence of simple gonorrhœa as distinguished from specific, and did not believe diagnosis between them easy.

*Dr. Cronyn* related a case similar to those related by Dr. Lockwood, wherein the woman was suffering from leucorrhœa. Had no doubt of the existence of simple gonorrhœa, but thought there was a great distinction between that and the specific disease in the relative severity of symptoms. Many symptoms of the specific disease are not present in the simple. The comparative readiness with which they yielded to treatment also furnished a ground of diagnosis. The difference between them was in the character of matter first applied.

*Dr. Miner* hardly believed a correct diagnosis could be formed, either from the severity of the symptoms or the results.

*Dr. White* was glad to see the gentlemen present so unanimous on the subject. For his part, he must say he knew of no means of discriminating between the two classes of cases. How did Dr. Cronyn wish to be reported?

*Dr. Cronyn* said authors told us to draw conclusions from the result of treatment. Did not wish to be understood as asserting that he could tell in every case; on the contrary, thought he could be easily imposed on. Thought on the whole the specific form was less amenable to treatment than the simple.

*Dr. Miner* said that whatever authors might say, or whatever the theoretical distinctions might be, it was practically impossible to make the distinction between these two forms of the disease. In doubtful cases patients should be allowed the benefits of the uncertainty.

*Dr. Jansen* thought the treatment would be mild or severe, according to the symptoms in any case. Did not believe it furnished any ground for diagnosis.

*Dr. Strong* said his experience agreed with that of others, as to the existence of the simple gonorrhœa, and he was very glad the subject had been brought up. Was of the opinion that specific gonorrhœa was more obstinate of treatment than the simple form.

*Dr. Wetmore* called attention to discharges from the vagina, which often followed diphtheria, not unlike that of gonorrhœa, diphtheria being a constitutional disease with local symptoms (as Prof. Rochester had always taught in his lectures) might affect any cavity lined with mucous membrane, and not involve the fauces at all. Hence it sometimes manifested itself in the vagina or urethra.

*Dr. Miner* thought the instances in which diphtheria attacked the vaginal or urethral cavities very rare indeed.

The prevailing diseases reported for the past month were typhoid and other low forms of fever, some measles, scarlatina, and roseola, and various diseases of the air passages, including diphtheritic croup.

*Dr. Wetmore* called attention to a remedy for the last named disease which he had found beneficial. It consisted n the vapor of carbonate of soda, obtained by putting one or two ounces into hot water, and enveloping the patient so he would have to inhale the vapor. It had suggested itself to him from hearing Dr. Moore say that this drug would in solution dissolve the membrane formed.

*Dr. White* thought the chief benefit arose from the vapor of the water which he had often tried.

Adjourned. JOSEPH A. PETERS,
*Secretary.*

## Figures and Tables

**Figure f1:**
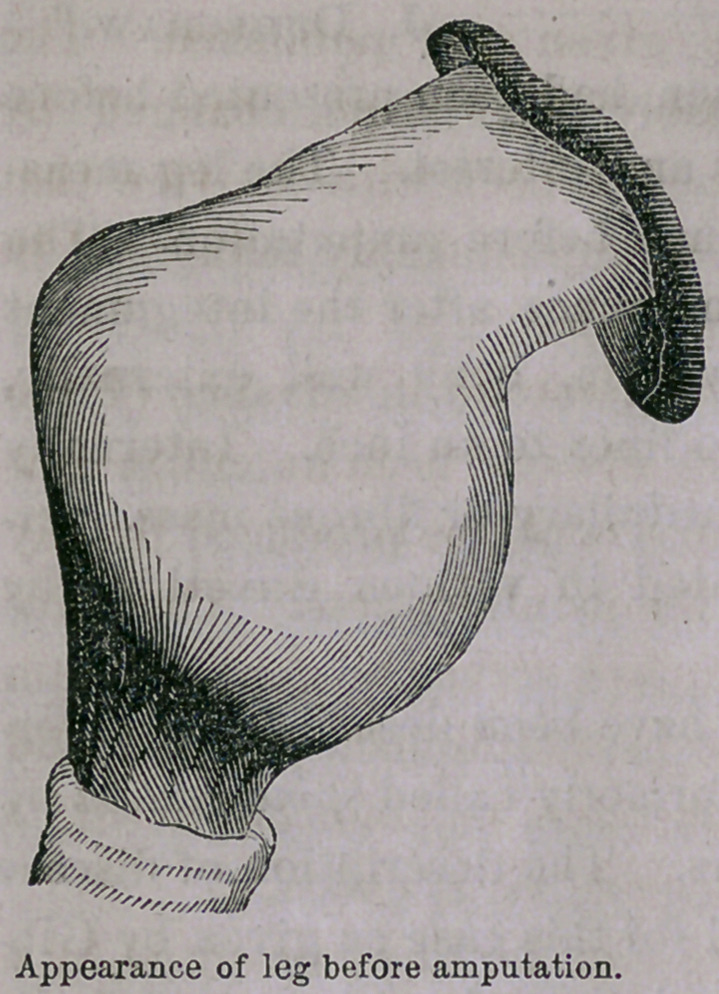


**Figure f2:**